# Assessment of Blood Lead Levels Among Children Aged ≤5 Years — Zamfara State, Nigeria, June–July 2012

**Published:** 2014-04-18

**Authors:** Muhammed Bashir, Nasir Umar-Tsafe, Kabiru Getso, Ibrahim M. Kaita, Abdulsalami Nasidi, Nasir Sani-Gwarzo, Patrick Nguku, Lora Davis, Mary Jean Brown

**Affiliations:** 1 Zamfara State Ministry of Health and Nigeria Field Epidemiology and Laboratory Training Program; 2 Nigeria CDC; 3 US CDC Country Office Nigeria; 4 Division of Emergency and Environmental Health Services, National Center for Environmental Health, CDC

Since 2010, Nigerian state and federal governments and the international community have been responding to an outbreak of lead poisoning caused by the processing of lead-containing gold ore in Zamfara State, Nigeria, that resulted in the deaths of approximately 400 children aged ≤5 years (1). Widespread education, surveys of high-risk villages, testing of blood lead levels (BLLs), medical treatment, and environmental cleanup all have been implemented. To evaluate the success of these remediation efforts in reducing the prevalence of lead poisoning and dangerous work practices, a population-based assessment of children’s BLLs and ore processing techniques was conducted during June–July 2012. The assessment found few children in need of medical treatment, significantly lower BLLs, and substantially less exposure of children to dangerous work practices. Public health strategies designed to identify and treat children with lead poisoning, clean up existing environmental hazards, and prevent children from being exposed to dangerous ore processing techniques can produce a sustained reduction in BLLs.

The 2010 outbreak of lead poisoning in Zamfara State caused by unsafe processing of lead-containing gold ore resulted in severe neurologic illness and death in children. When processed dry with low technology methods, the gold ore produced fine particles that contaminated water and food crops and were easily inhaled or ingested during normal hand-to-mouth activities common among toddlers (2).

When inhaled or ingested, lead can cause damage to the brain, kidneys, bone marrow, and other body systems in young children. In infants and children, BLLs as low as 5 *μ*g/dL have been associated with developmental problems, including impaired cognitive function, behavioral difficulties, impaired hearing, and reduced stature (3). BLLs ≥75*μ*g/dL can cause coma, convulsions, and death. No safe BLL has been identified for children (4).

During June–July 2012, the Zamfara State Ministry of Health, Nigerian Field Epidemiology and Laboratory Training Program, and CDC collaborated to conduct a representative, cross-sectional, population-based, multistage stratified cluster design survey to estimate the geometric mean (GM) BLLs of children living in Zamfara State aged ≤5 years. Investigators also examined the extent of exposure to ore processing methods that generate dust (i.e., crushing ore, dry grinding ore using power flour grinders, and open air drying of ore), and they examined the ore processing practices among the children’s mothers, an important factor because ore processing inside the family compound is a female role in this population. Because mothers also are responsible for child care, ore processing among mothers is a risk factor for young children who eat, sleep, and play within the compound.

To create systematic samples for village-level and family-level surveys, a total of 112 villages initially were selected from among the 14 local government areas in Zamfara State, proportionate to the population of the areas, using ambient population distribution software (5). An additional 10 villages were selected as potential replacements should some selected villages be inaccessible or unsafe, bringing the total to 122. To obtain population-based estimates of children’s blood and environmental lead levels, one child in each of seven systematically selected families was surveyed in more detail from each of 56 villages systematically selected from among the 122 villages, for a total of 392 children in the study sample.*

Venous blood samples were collected from children aged ≤5 years. BLLs were analyzed using inductively coupled plasma mass spectroscopy, with a lower limit of detection of 0.25 μg/dL. The precision and quality assurance measures used have been described previously (6). GM BLLs were analyzed for correlation with exposure to different ore processing methods, with statistical significance determined at p<0.05. The study protocol was approved by the National Health Research Ethics Committee of Nigeria and the CDC Institutional Review Board.

Among the 392 children aged ≤5 years, the mean age was 35 months. The mothers of 69 (17.6%) children were involved in ore processing activities. Sixty-one (15.6%) mothers used processing techniques that generated large amounts of dust, and 17 (4.3%) mothers processed ore using dust-generating techniques within the family compound.

A history of convulsions was reported in 90 (23.0%) children. Thirty-four of the children lived in villages that used dust-generating ore processing methods, 40 lived in villages that used non–dust-generating methods, and 16 lived in villages that did not do ore processing (Table).

Venous blood samples were collected from 383 (97.7%) of the 392 children. The GM BLL for the 383 children was 6.9 *μ*g/dL (range = 1.6–61.0 *μ*g/dL). A total of 88 children (23.0%) had BLLs ≥10 *μ*g/dL, and four (1.0%) had BLLs ≥45 *μ*g/dL, the threshold for treatment with chelation therapy recommended by CDC (7).

The GM BLL of the children whose mothers were involved in dust-generating ore processing activities was 8.5 *μ*g/dL (standard deviation [SD] = 11.1), compared with 6.4 *μ*g/dL (SD = 5.3; p<0.001) among those whose mothers used non–dust-generating ore processing methods (Table). When dust-generating activities were conducted within the family compound, the GM BLL in children was 8.9 *μ*g/dL (SD = 9.8). All four children with BLLs ≥45 *μ*g/dL had mothers involved in dust-generating activities within the family compound.

Although the use of mercury for gold extraction to amalgamate gold was not defined as a dust-generating activity, children living in villages where mercury was used had BLLs significantly higher than children where gold ore was not processed (GM 11.2 *μ*g/dL [SD = 5.1] compared with 3.4 *μ*g/dL [SD = 5.1], p<0.05). Additionally, for children living in villages where excess mercury was burned off the ore, the GM BLL tended to be significantly higher (GM 13.1 *μ*g/dL [SD = 16.6]), compared with children living in villages in which mercury was not used to amalgamate gold.

## Discussion

The 2010 outbreak of lead poisoning in Zamfara State was unprecedented in recent decades. In two investigations in 2010 conducted to identify villages where risk for lead poisoning was extremely high, one found that 97% of children had BLLs ≥45 *μ*g/dL, and the second found that, among ore processing villages, 30% of children had BLLs ≥45 *μ*g/dL, but no children in villages that did not process ore had BLLs this high. (8)

Since 2010, a team of international, Nigerian federal, and Nigerian state public health agencies, environmental remediation specialists, health-care providers, and educators has worked to reduce BLLs in young children. These efforts have resulted in identification of lead contamination in approximately 50 villages, cleanup of environmental lead contamination in 11 villages, chelation therapy for children with BLLs ≥45 *μ*g/dL, widespread public education campaigns, and training for local workers responsible for testing and remediation activities (9).

In the assessment described in this report, 74% of the 56 villages were involved in the gold trade, and the GM BLL was significantly higher among children whose mothers processed ore using dust-generating methods. Few families (20 [5%] compared with 84 [71%] in 2010) (1) were processing ore within the family compound, where children aged ≤5 years spend most of their time, and three of the 20 families used non–dust-generating ore processing methods. However, the highest GM BLLs were found among children who lived in villages where mothers used certain non–dust-generating methods, such as amalgamating ore using mercury or burning excess mercury off the ore; the high GM BLLs might have resulted from toxic lead fumes released during burning.

Although work remains, much has been done to address the problem of lead exposure in Zamfara State. New and safer processing techniques that control dust and residual ore wastes, a better understanding of potential exposure to lead-contaminated foodstuffs, continued BLL surveillance, chelation therapy when warranted, and environmental cleanup of hazardous sites remain critical (9). When such strategies are successfully implemented, a sustained reduction of BLLs in children can be achieved.

What is already known on this topic?

Processing of lead-containing gold ore using dust-generating methods caused the deaths of approximately 400 children from lead poisoning in Zamfara State, Nigeria, in 2010 and left thousands of others with severe disabilities. The Nigerian and Zamfara State governments, in collaboration with international organizations including CDC, have been working to identify and treat affected children, clean up the hazardous processing sites, and educate the community about the dangers of lead.

What is added by this report?

This assessment found that most families are using safer ore processing methods and blood lead levels among young children are lower than those found in 2010. Only 20 families (5%) were processing ore within the family compound, where children aged ≤5 years spend most of their time, compared with 84 families (71%) in 2010.

What are the implications for public health practice?

Collaboration between governments and the international community can prevent lead poisoning in children. When strategies such as use of processing techniques that control dust and residual ore wastes, continued blood lead surveillance, chelation therapy when warranted, and environmental cleanup of hazardous sites are successfully implemented, a sustained reduction of blood lead levels in children can be achieved.

## Figures and Tables

**TABLE:** Selected characteristics among 392 children aged ≤5 years living in 56 villages in an area where in 2010 fatal lead poisoning was linked to processing gold ore, by village ore activities — Zamfara State, Nigeria, 2012

	Living in villages that use dust-generating ore processing* (n = 108)		Living in villages that use non–dust-generating ore processing† (n = 193)		Living in villages that do no ore processing§ (n = 91)		
				
Characteristic	No.	(%)	No.	(%)	No.	(%)	p-value
**Sex**							
Male	50	(46.3)	109	(56.5)	44	(48.4)	
Female	57	(52.8)	83	(43.0)	43	(47.4)	
No response	1	(0.9)	1	(0.5)	4	(4.4)	0.05
**Age group (mos)**							
<12	5	(4.6)	12	(4.2)	9	(9.9)	
12–23	21	(19.4)	47	(16.7)	14	(15.4)	
24–35	14	(13.0)	47	(16.7)	14	(15.4)	
36–47	23	(21.3)	60	(21.1)	21	(23.1)	
48–60	42	(38.9)	105	(37.0)	28	(30.8)	
No response	3	(2.8)	13	(4.6)	5	(5.5)	0.18
**History of convulsions**							
Yes	34	(31.5)	40	(20.7)	16	(17.6)	
No	72	(66.7)	150	(77.7)	71	(78.0)	
No response	2	(1.9)	3	(1.6)	40	(4.4)	<0.001
**Mother processes ore**							
Yes	61	(56.5)	6	(3.1)	2	(2.2)	
No	46	(42.6)	179	(92.1)	85	(93.4)	
No response	1	(0.9)	8	(4.2)	4	(4.4)	<0.001
**Mother processes ore within the family compound**							
Yes	17	(15.7)	3	(1.2)	0	(0)	
No	91	(84.3)	190	(98.5)	91	(100.0)	<0.001
**Ore processing performed outside the family compound**							
Yes	40	(37.0)	3	(1.6)	3	(3.3)	
No	68	(63.0)	190	(98.1)	88	(96.7)	<0.001
**GM BLL (** ***μ*** **g/dL) in children (n = 383)**	**GM BLL**	**(SD)**	**GM BLL**	**(SD)**	**GM BLL**	**(SD)**	**p-value**
	
	8.5	(11.1)	6.4	(5.3)	3.4	(5.1)	<0.001

**Abbreviations:** GM BLL = geometric mean blood lead level; SD = standard deviation.

* Dust-generating activities include breaking ore, grinding ore, and drying ore.

† Non–dust-generating activities include washing ore and mercury amalgamation.

§ Defined as no ore processing within 1.26 mi (2 km) of the village.

## Footnotes

* A sample size of 46 villages had been determined large enough to estimate the prevalence of elevated BLLs ≥10 *μ*g/dL (then defined as the CDC BLLs of concern). An additional 10 villages were added to the sample in the event some of the 46 selected villages were inaccessible or unsafe, bringing the total to 56 villages, each with seven children, for a total of 392 children in the sample.

## References

[1] Abdelaziz S (2010). Aid groups say lead poisoning has killed 400 children in Nigeria. CNNWorld.

[2] Dooyema CA, Neri A, Lo YC (2012). Outbreak of fatal childhood lead poisoning related to artisanal gold mining in northwestern Nigeria, 2010. Environ Health Perspect.

[3] Agency for Toxic Substances and Disease Registry (2007). Toxicological profile for lead.

[4] Lanphear BP, Hornung R, Khoury J (2005). Low-level environmental lead exposure and children’s intellectual function: an international pooled analysis. Environ Health Perspect.

[5] Oak Ridge National Laboratory Geographic information science and technology: LandScan.

[6] CDC (2009). Blood cadmium and lead using ICPMS. NHANES 2009–2010.

[7] CDC (2002). Managing elevated blood lead levels among young children: recommendations from the Advisory Committee on Childhood Lead Poisoning Prevention.

[8] Lo YC, Dooyema C, Neri A (2012). Childhood lead poisoning associated with gold ore processing: a village-level investigation—Zamfara State, Nigeria, October–November 2010. Environ Health Perspect.

[9] Medecins Sans Frontieres (2012). Time is running out: Zamfara State lead poisoning crisis.

